# What Matters Most in Life? A German Cohort Study on the Sources of Meaning and Their Neurobiological Foundations in Four Age Groups

**DOI:** 10.3389/fpsyg.2021.777751

**Published:** 2021-11-30

**Authors:** Christopher Karwetzky, Lena Werdecker, Tobias Esch

**Affiliations:** Faculty of Health, Institute for Integrative Health Care and Health Promotion, Witten/Herdecke University, Witten, Germany

**Keywords:** sources of meaning, meaningfulness, neurobiology, motivation, aging

## Abstract

Existing work in the field of positive psychology suggests that people can draw meaning from a variety of sources. The present study aimed to identify the most important sources of meaning and to explore the role of age and neural adaptation processes in this context. As part of a large German cohort study, 1,587 individuals between 12 and 94 years were asked to provide a maximum of five responses to the question “What matters most to you in life?” We divided the study population into four age groups and analyzed the obtained answers qualitatively and quantitatively using (1) word clouds and (2) frequency comparisons based on a summarizing content analysis. A chi-squared test was used to test the observed differences between age groups. Identified sources of meaning could be clustered into 16 main and 76 subcategories, with *relationships* (by 90% of respondents) and *health and well-being* (by 65% of respondents) being the most frequently named main categories, followed by a *good living environment* (by 28%), *(leisure) time* (by 26%), and *work* (by 24%). The study revealed some remarkable age-related patterns. While the importance of *partnership* increased with age, *social networks* were less important to older individuals. We also found that, for example, the importance of *self-realization*, *success and career* decreased with age, while the opposite was true for *life satisfaction* and *peace and harmony*. *Security* was most important to individuals in the two middle age groups between 30 and 69 years. The study advances our understanding of meaning across various ages by showing that individuals of different ages perceive different things as meaningful to them. Interpreting our results in the light of a neurobiological model of motivation systems, we argue that neural adaptation processes may play an important role in the (changing) perceptions of meaning throughout life.

## Introduction

Earliest records teach us that the pursuit of a good and meaningful life has always been an important aspiration of mankind. But what aspects enrich our lives and fill them with meaning? What matters most? The research on meaning in life addresses these questions, defining meaning as a construct of human experience that provides life with direction through coherence, purpose, and significance (Martela and Steger, 2016). It is influenced by multiple factors including, but not limited to, achievements, relationships, work, religion, spirituality, self-transcendence, generativity, personal growth, leisure activities, traditions, and values (Reker and Woo, 2011; Schnell, 2020). Findings from different countries indicate that perceptions regarding meaning in life have a cultural component. For example, in Denmark, generativity was ranked as a primary source of meaning (Pedersen et al., 2018), whereas in a Brazilian study the most important source of meaning was attributed to religion (Damásio et al., 2013). By contrast, human relationships (family/interpersonal relations) were at the foreground of meaning in studies from New Zealand (Groudan and Jose, 2014), the United States (De Vogler and Ebersole, 1981; Baum and Stewart, 1990), and the Netherlands (Debats, 1999). Furthermore, the perception of meaning in life also correlates with important psychological factors such as health, anxiety/hypochondria (Yek et al., 2017), cognition (Aftab et al., 2019), and depression (van der Heyden et al., 2015; Volkert et al., 2019).

Findings on the relationship between meaning and age are not conclusive. While some authors report higher levels of meaning in older people (Schnell and Becker, 2007; Steger et al., 2009), others found an inverted U-shape with a peak around age 60 (Aftab et al., 2019). Interestingly, empirical research also points to age-related differences regarding the sources of meaning. While younger adults tend to draw meaning from the achievement of personal goals, self-realization, or the fulfillment of basic needs, older individuals draw meaning from spirituality, engagement in society, traditions, and self-transcendence (Reker et al., 1987; Reker and Wong, 1988; Hupkens et al., 2018). It is unclear whether the observed changes form part of a gradual, ongoing process, or occur in response to particular life experiences (or in certain time intervals) as suggested by Alter and Hershfield (2014). One potential explanation for the changed perception regarding the source of meaning is due to adjustments in life goals throughout the life span (van Rast and Marcoen, 2000). The adjustment of life goals entails a different perception of—and ability to distinguish—between realistic and unrealistic goals, depending on learning processes and the expectation of the number of years of life remaining (Argyle, 1999; Carstensen et al., 1999; Brandtstädter, 2015).

Although empirical research has significantly improved our understanding of meaning in life, there is still no definitive picture of its sources, possible changes in perception regarding these sources across different age groups, and the underlying physiological processes related to this. This article seeks to better understand the sources of meaning and therefore provide valuable insights for society but also for each individual.

### Research Objectives

By evaluating data from a large German cohort study, we attempted to identify and quantify the sources of meaning in four age groups. We hypothesized that specific factors determine the perception of meaning in life and that these determinants vary across age groups. The results are discussed in the light of a *neurobiological model of motivation systems*, a model that relates perceptions of a “good life” to lifelong neurophysiological growth and maturation processes. This article is part of a larger study on the determinants of happiness, life satisfaction, and meaning.

## Materials and Methods

### Participants

Our analyses are based on a survey conducted in Germany between September 2017 and January 2018. In total, 1,587 people aged between 12 and 94 participated in the study.

The distribution of our sample, comprising both males and females, across four age groups is shown in Table 1.

**TABLE 1:** Study population and distribution across different age groups.

	Total	Age group 1 (≤29 years)	Age group 2 (30 to 49 years)	Age group 3 (50 to 69 years)	Age group 4 (≥70 years)
Study participants	1,587 (100%)	367 (23%)	552 (35%)	506 (32%)	162 (10%)
Male	587 (37%)	130 (36%)	173 (31%)	181 (36%)	103 (64%)
Female	1,000 (63%)	237 (64%)	379 (69%)	325 (64%)	59 (36%)

For cross-generational comparisons, we defined four age groups:

Age group 1: up to 29 years
Age group 2: 30 to 49 years
Age group 3: 50 to 69 years
Age group 4: 70 years or older

The age groups should reflect typical phases of life, such as youth and education (Age group 1), starting work, building a career and establishing a family (Age group 2), middle adulthood (Age group 3), and retirement (Age group 4).

The sample largely represents Germany’s actual demographic distribution. Participants in Age group 2 were slightly overrepresented (35 vs. 28%), while those in Age group 4 were slightly underrepresented (10 vs. 17%; Federal Statistical Office, 2021). Overall, more females (1,000) than males (587) participated in the study, with some differences between age groups.

### Survey Instrument

Data was collected using both online and paper questionnaires. The questionnaire began with the question “What matters most to you in life?”. Participants could give a maximum of five answers. No prioritization was asked for, and no word limit was set for the responses.

Furthermore, we queried various socio-demographic variables, such as age, subjective health status, financial worries, occupational status, or city size although not all collected variables were relevant for this part of the study. To ensure the questionnaire’s comprehensibility, we pre-tested it with 15 individuals in August 2017.

### Sampling and Recruitment

The relevant population comprised all persons aged 10 years and older living in Germany. Individuals with cognitive impairments (e.g., dementia) were excluded from participation. We shared information about the possibility to participate in the survey *via* radio (Deutschlandfunk), television (WDR), social media (Facebook), and the Witten/Herdecke University’s webpage. In addition to an online survey (1,027 participants), 560 people were recruited in various public settings (e.g., cafés, trains), four general practitioners’ and ophthalmologists’ practices, and two schools. All study participants received extensive information material on the study objectives and the processing of the data collected.

The study obtained ethics approval from the Witten/Herdecke University’s Ethics Committee (ethics vote no. 138/2017).

### Analysis

A two-stage procedure was selected to answer the research questions. First, we generated word clouds to graphically analyze the unprocessed material. Articles, pronouns, and adverbs were excluded from the word clouds to focus on content-bearing words.

**FIGURE 1:**
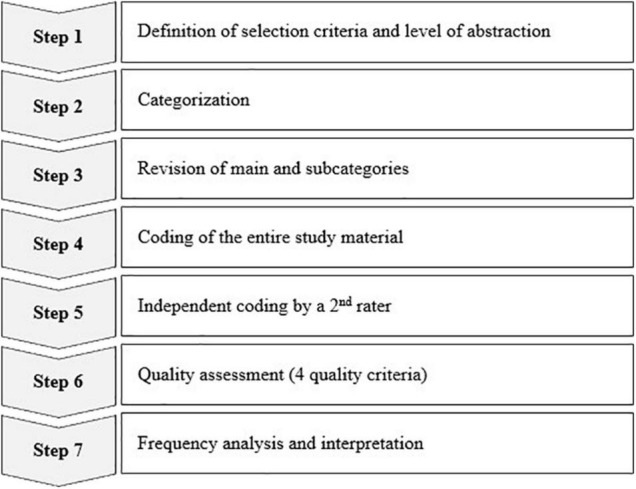
Schematic overview of the qualitative content analysis approach.

Subsequently, we conducted a summarizing content analysis according to Mayring (2000) to investigate the answers in a structured way. All analyses were carried out in MAXQDA 2020 (Kuckartz and Rädiker, 2019). Methodologically, the content analysis followed comprehensive rules, which we defined in advance using a 7-step process model (see Figure 1).

Frequencies were first calculated for the main and then for the subcategories. To analyze the share of participants that mentioned a particular category, we divided the number of codings per category by the number of participants. In cases where participants gave several answers that could be assigned to the same main or subcategory, we counted only one coding per category. We used the chi-squared test to analyze differences between age groups and considered *P* ≤ 0.05 statistically significant.

In total, we received 6,609 responses from 1,587 respondents, which corresponds to an average of 4.2 responses per participant. However, some differences were observed in the distribution of responses by age groups. In Age groups 1–3, the average number of responses ranged from 4.1 to 4.4, while in Age group 4 it was only 3.3.

All analyses were conducted in German. We then translated the results into English.

## Results

### Word Clouds

**FIGURE 2:**
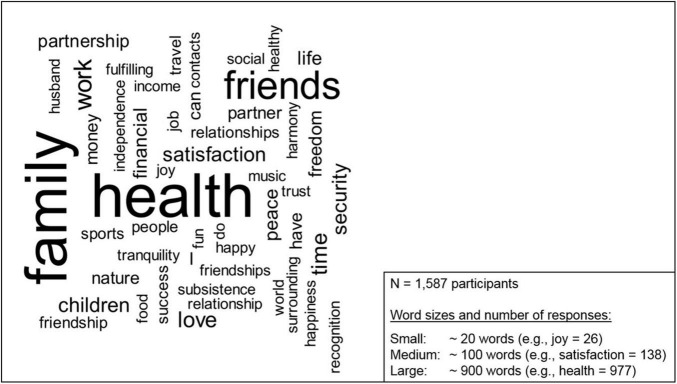
Word cloud representing the distribution of responses from the overall study population.

For all participants across the various age groups, *health*, *family*, and—by some distance—*friends* were mentioned most frequently as being most important to them in life. The next most frequently mentioned group of terms comprised *work*, *love*, *time*, and *children*. As shown in Figure 2, *money* did not feature as often as could be expected among the 50 most frequently used words to describe factors that contribute meaning to life.

A comparison of the responses from the youngest and the oldest age group is shown in Supplementary Figure 1. We observed that *health* and *family* were mentioned very often in both age groups. Interestingly, *friends*, *work*, and *time* were mentioned more often by younger individuals, whereas, for example, *partnership* and *peace* were mentioned more often by older respondents.

### Summarizing Content Analysis

The summarizing content analysis resulted in 16 main and 76 subcategories. We present the overall categorization including exemplary responses in Supplementary Table 1.

The frequencies at the **main category level** for the overall study population and four age groups are shown in Table 2. We sorted the categories in descending order according to the frequency with which topics that could be allocated to them were mentioned in the overall study population (column “Total”).

**TABLE 2:** Distribution of responses across the main categories for the overall study population and according to different age groups.

	Main categories	Total^1^	Age group 1	Age group 2	Age group 3	Age group 4	χ^2^	(df)	*P*
**	Relationships	90%	93%	91%	87%	85%	13.82	(3)	0.003
***	Health and well-being	65%	50%^b^	65%	71%	76%	52.84	(3)	<0.001
***	Good living environment	28%	17%^b^	24%	38%^a^	36%	55.83	(3)	<0.001
***	(Leisure) time	26%	28%	31%^a^	24%	10%^b^	21.19	(3)	<0.001
***	Work	24%	31%^a^	27%	22%	4%^b^	51.29	(3)	<0.001
***	Security	18%	10%^b^	20%	24%^a^	10%^b^	40.43	(3)	<0.001
***	Personal growth	14%	18%^a^	16%	9%^b^	7%^b^	24.39	(3)	<0.001
***	Sense and meaningfulness	12%	8%^b^	15%	14%	8%	12.42	(3)	<0.001
***	Happiness	11%	16%^a^	11%	9%	4%^b^	20.59	(3)	<0.001
	Values	10%	10%	11%	11%	10%	0.09	(3)	0.993
***	Freedom	10%	7%^b^	15%^a^	9%	6%	23.58	(3)	<0.001
	Life satisfaction	9%	10%	8%	9%	12%	1.79	(3)	0.616
*	Material possessions	9%	11%	8%	9%	3%^b^	9.58	(3)	0.023
	Social rank	4%	4%	4%	5%	2%	2.66	(3)	0.448
	Other	4%	5%	4%	3%	2%	5.07	(3)	0.167
	Long life	0%	0%	0%	1%	1%	4.49	(3)	0.213

	Codings per participant	4.2	4.1	4.4	4.2	3.3			
	N	1,587	367	552	506	162			

*^1^Table is sorted in descending order in column “Total”.*

*^a^Standardized residuals ≥ 2.*

*^b^Standardized residuals ≤ −2. Significance levels (Pearson’s chi-squared test):*

*P < 0.05; **P < 0.01; and ***P < 0.001.

In terms of all respondents, 90% considered *relationship*-associated aspects to be very important. *Health and well-being* were placed second with 65%, and approximately a quarter of respondents mentioned aspects related to a *good living environment* (28%), *(leisure) time* (26%), and *work* (24%).

When comparing the responses obtained from different age groups, we observed some significant differences. Although *relationships* remained at the top of the list in all age groups, their frequency decreased from 93% in Age group 1 to 85% in Age group 4, whereas *health and well-being* and a *good living environment* were mentioned more frequently by older than by younger participants.

Additionally, the analysis also showed some interesting age-specific differences regarding less frequently mentioned aspects. For example, *Personal growth* was particularly important for younger people (Age group 1), while *security* and *sense and meaningfulness* were mentioned most often by Age groups 2 and 3.

Some other notable variations were observed for the main categories *happiness*, *life satisfaction*, and *freedom*. While 16% of the participants in Age group 1 mentioned aspects related to momentary *happiness*, this proportion fell to only 4% in Age group 4. Although *freedom* was regarded as being very important by 15% of respondents in Age group 2, this perception was shared less frequently by those in Age group 3 (9%), and Age group 4 (6%). *Material possessions* were mentioned significantly less often in Age group 4 (3%) than among younger participants.

The **frequencies at the subcategory** level are shown in Table 3. The categories were again sorted in descending order according to their frequency in the responses of the overall study population (column “Total”). To facilitate readability, the table only includes subcategories that contain codes from at least 5% of the study participants in at least one age group, while the frequency of the remaining subcategories is presented in aggregated form (“Total other subcategories”). Only 12% of the survey respondents mentioned any of these remaining subcategories, indicating that most codings are covered by the displayed categories.

**TABLE 3:** Distribution of responses across the subcategories for the overall study population and according to different age groups.

	Subcategories	Main categories	Total^1^	Age group 1	Age group 2	Age group 3	Age group 4	χ^2^	(df)	*P*
**	Family	Relationships	68%	72%	70%	66%	57%	13.12	(3)	0.004
***	Health for me	Health and well-being	61%	49%^b^	60%	67%	72%	38.03	(3)	<0.001
***	Social network	Relationships	44%	60%^a^	42%	39%	27%^b^	61.75	(3)	<0.001
**	Partnership	Relationships	18%	14%	19%	17%	26%^a^	11.83	(3)	0.008
***	Financial security	Security	16%	7%^b^	18%	22%^a^	9%^b^	42.74	(3)	<0.001
***	Love and trust	Relationships	15%	21%^a^	17%	12%	8%^b^	20.65	(3)	<0.001
***	Peace and harmony	Good living environment	13%	6%^b^	9%^b^	19%^a^	22%^a^	54.94	(3)	<0.001
	Activity/entertainment hobbies	(Leisure) time	12%	16%	11%	13%	9%	6.56	(3)	0.087
***	To have time and spend it with what/those you love	(Leisure) time	10%	9%	15%^a^	7%	1%^b^	35.91	(3)	<0.001
	Satisfaction for me	Life satisfaction	9%	9%	8%	8%	11%	1.71	(3)	0.635
***	Nature	Good living environment	8%	3%^b^	7%	13%^a^	7%	32.69	(3)	<0.001
***	Happiness for me	Happiness	8%	14%^a^	8%	5%^b^	2%^b^	31.44	(3)	<0.001
***	Self-realization	Personal growth	6%	8%	8%	4%^b^	2%^b^	17.21	(3)	<0.001
**	Health for us or others	Health and well-being	6%	2%^b^	7%	7%	8%	13.62	(3)	0.004
*	Money	Material possessions	5%	7%	5%	5%	1%^b^	8.20	(3)	0.042
	Societal security	Good living environment	5%	6%	5%	4%	2%	2.86	(3)	0.414
***	Success and career	Work	4%	11%^a^	3%	2%^b^	0%^b^	52.56	(3)	<0.001
*	AE: Fulfilment	Sense and meaningfulness	4%	4%	6%^a^	3%	2%	9.47	(3)	0.024
*	Holidays/travel	(Leisure) time	4%	4%	6%	4%	1%^b^	9.02	(3)	0.029
	Self-determination	Freedom	4%	2%	5%	5%	4%	5.56	(3)	0.135
	Recognition	Social rank	4%	4%	4%	5%	2%	2.75	(3)	0.431
	New experiences and challenges	Personal growth	4%	5%	5%	2%	3%	6.39	(3)	0.094
	Joy and satisfaction at work	Work	4%	4%	5%	3%	1%^b^	7.19	(3)	0.066
	A society worth living in	Good living environment	4%	2%	3%	5%	3%	4.26	(3)	0.235
**	Faith and spirituality	Sense and meaningfulness	3%	2%	2%	6%^a^	4%	12.57	(3)	0.006
	*Total other subcategories*		*12%*	*12%*	*12%*	*13%*	*11%*			

	Codings per participant		4.2	4.1	4.4	4.2	3.3			
	N		1,587	367	552	506	162			

*^1^Table is sorted in descending order in column “Total” and contains only subcategories that have a frequency ≥ 5% in at least one age group.*

*^a^Standardized residuals ≥ 2.*

*^b^Standardized residuals ≤ −2. Significance levels (Pearson’s chi-squared test):*

*P < 0.05; **P < 0.01; and ***P < 0.001.

Within *relationships*, which was the most important main category, *family* (68%), *social network* (44%), and *partnership* (18%) were the most frequent subcategories. When comparing the responses between age groups with increasing age, the frequency of responses involving *family* and, in particular, *social network* decreased, while a *partnership* was mentioned more often by older than by younger people. In the main category *health and well-being*, which was the second most important category at the upper level, both *health for me* and *health for us or others* appeared to be more important for older than for younger individuals. Although *financial security* was important to 18% of respondents in Age group 2 and 22% in Age group 3, interestingly the values were much lower for Age group 1 (7%) and Age group 4 (9%). Aspects related to *peace and harmony* were mentioned more frequently by older participants, as was shown by the difference between the responses of Age group 1 (6%) and Age group 4 (22%). The opposite was observed in terms of *success and career*, as aspects of this subcategory were mentioned more frequently by respondents in Age group 1 (11%) than by respondents in subsequent age groups (3% or less). Overall, aspects that are more material or performance-oriented such as *money*, *self-realization*, and *new experiences and challenges* were mentioned less often by older participants.

## Discussion

### Interpretation of Key Findings

The study aimed to identify and compare the perceived sources of meaning in a randomly selected sample of persons in Germany, and to determine the differences in these perceptions across four different age groups. By examining the categorization derived from the data, the large number of categories identified (16 main and 76 subcategories) indicates that, for the sample in question, meaning can be drawn from many sources. Despite the relatively large number of categories, the analysis showed that *relationships* (especially *family*) were overwhelmingly ranked as being the most important source of meaning, followed by *health and well-bein*g. Further aspects such as a *good living environment*, which includes the subcategories *peace and harmony*, *societal security*, and *environmental protection*, as well as *(leisure) time* were also indicated as being important sources of meaning, although not nearly as important as *relationships*. Toward the middle of the range of the frequency of responses, aspects related to *work* and *success* were mentioned, alongside *freedom* or *personal growth*. *Material possessions* or *social rank* were of great importance to relatively few people.

When comparing the results between the four predefined age groups, we found some interesting differences, such as the higher level of importance attributed to *partnership*, *peace and harmony*, and a lower level of importance attributed to *social networks, success and career*, and *self-realization* in older people.

The **neurobiological model of motivation systems** (Esch, 2017; Michaelsen and Esch, 2021) hypothesizes that many of these changes can, at least partly, be attributed to age effects. According to this model, our perceptions of meaning and well-being are subject to lifelong neurophysiological processes of growth, pushing maturation and translating experiences into neuronal structures (“neuronal plasticity”). The model distinguishes three motivation systems (A, B, and C), that are ultimately aligned under the primary goal of optimally adapting to our socio-cultural environment throughout our lives.

The Wanting System (also “Type A Motivation”) characterizes the first phase of life including young adulthood. To adequately capture this phase of life in our analyses, we broadly clustered Age group 1 up to age 29. It is clear that this is a broad clustering of several phases (childhood, adolescence, and early adulthood) into one broad phase. However, it is worth noting that according to the model, these phases share similar neurobiological characteristics, i.e., great potential for freedom and adaptation, and incomplete neural preparation for the concrete challenges of life. Under the strong influence of dopamine, peak moments are perceived as a thrill or “happiness.” Against the background of Type A Motivation, it seems plausible that *success and career* could be identified as important sources of meaning in life in Age group 1. Similarly, factors such as *social network*, *material possessions*, *happiness for me*, *leisure time*, *self-realization* and *success, and career* were named more often by respondents in this age group than by older respondents.

The Threat-Avoidance System (also “Type B Motivation”) assumes that our body activates its stress physiology more often as we adapt and mature. This correlates to the observation that in midlife, many people prefer to “persist and defend” rather than to “conquer,” because they increasingly crave security and try to avoid stress (see also Stefano et al., 2005). In the results of our survey, this increased need for safety and protection was apparent from the responses of Age groups 2 and 3, which attributed increasing importance to *financial security* and a decreased importance to categories such as *freedom* or *success and career*.

Finally, the Non-Wanting-System (“Type C Motivation” or “Quiescence”) is characterized by altruism and affiliation. If individuals adapt to the circumstances of life and mature, lasting satisfaction, inner serenity, and self-knowledge (“wisdom”) can increasingly develop with age. Our results showed some patterns that could indicate the development of Type C Motivation in the Age group 4 respondents. Firstly, according to the neurobiological model, experiences (and stress) lead to an adjustment of aspirations and a focus on the essential. This could be the reason why the respondents in Age group 4 on average provided a lower number of sources of meaning (3.3/5 possible responses) as opposed to respondents from other age groups (4+/5 possible responses). Furthermore, the increased importance attributed to inner *peace and harmony* and *partnership* by respondents in Age group 3 and Age group 4, corresponding with the decreased importance attributed to *self-realization* and *money* for Age groups 2–4 as a factor of increasing age, suggest an increasing development away from a Type B toward Type C motivation.

An overview of the *neurobiological model of motivation systems*, including involved brain areas, is presented in Supplementary Figure 2.

In general, we interpret the high number of responses across all age groups pertaining to *family* to indicate the significance of generativity in people’s lives. This finding is consistent with those from the study conducted in Denmark by Pedersen et al. (2018) and Schnell’s research involving people from different countries, age groups, and psychological conditions (Schnell, 2020). According to the neurobiological model, the transmission of experiences, knowledge, and cultural heritage to subsequent generations (“generativity”) is of great importance, especially in later years, which could explain the positive correlation that some researchers have found between grandparenting, perceived meaning, and subjective well-being (e.g., Park, 2018).

While the significance of relationships for meaning was apparent for all age groups, it was interesting to note a difference in emphasis regarding relationship types among the different age groups surveyed. In our results, the importance attributed to *social networks* decreased in Age group 4, while *partnership*-related aspects were regarded as becoming increasingly important with increasing age. It thus appears that the need for social interaction shifts from having a large circle of friends in younger years to the core of *partnership* and *family* as people grow older.

Regarding the importance of *health and well-being*, our analyses showed a gradual increase in the number of responses with increasing age. This was an interesting finding since our quantitative analyses of the same database showed that the correlation between health and life satisfaction was weaker among older than younger individuals,^1^. Other authors have controversially discussed the relationship between health and subjective well-being (e.g., Okun et al., 1984; Berg et al., 2006; Gana et al., 2013; Steptoe et al., 2015), with several studies pointing toward a “health paradox.” Although the importance of health for meaning and well-being tends to increase with increasing age, only a few illnesses are statistically associated with lower levels of subjective well-being (Berg et al., 2009, 2011; Hanson et al., 2019; Näsman et al., 2020). According to the neurobiological model, physical health is especially important to experience happiness driven by the Wanting System (Type A Motivation), which is typically seen during adolescence and young adulthood. As we grow older and mature, a certain level of physical discomfort and health problems can be regarded as “part of life” and thus be perceived as being less of an impairment to subjective well-being.

Finally, *faith and spirituality*, which have been identified as very important sources of meaning in other countries (e.g., Brazil and Italy) and especially among older individuals (e.g., Damásio et al., 2013; Manuti et al., 2016), did not play a significant role in this study. This difference could potentially be attributed to cultural and/or socio-economic differences. However, according to the sociodemographic data collected, the proportion of religious or faithful individuals increased from age group to age group; reaching 63% in the oldest group. In the youngest age group, it was only 29%.

From a societal perspective, this study has a number of practical implications and stimulates further thought in a variety of ways. The perception of older people as important contributors to our cultural heritage, the widespread goal of eternal health and youth, and the extraordinary importance of community and connection for each individual but also our society as a whole (e.g., Costabile et al., 2021) are just a few examples.

### Strengths, Limitations, and Outlook

The present study is characterized by strengths, but also limitations, that should be considered when interpreting the results.

To assess the quality of our category system, a second rater re-coded the responses of 100 participants. Cohen’s Kappa (Cohen, 1960), which was used as a measure of agreement between the first and the second coding, had a value of 0.97. This value points to a high quality of the category system and the overall coding, as values between 0.61 and 0.80 are usually classified as *substantial*, and values between 0.81 and 1.00 as *almost perfect* (Fleiss, 1981). Furthermore, the 16th main category *other* accounted for only 0.95% of all codings, indicating that almost all mentions were covered by the 15 content-bearing categories. Finally, the study had a sample size that is substantial compared to most other qualitative studies on the sources of meaning (e.g., Delle Fave et al., 2013; *N* = 666; Groudan and Jose, 2014; *N* = 247).

Regarding the limitations, the use of cross-sectional instead of longitudinal data should be highlighted. Instead of following the same people over a longer period, we surveyed individuals of different ages and conducted frequency comparisons across age groups. Although the use of cross-sectional data is common due to time constraints, it is important to understand that age effects are only one possible explanation for the patterns observed. Secondly, the study is limited by some inequalities in the sample, namely the unequal gender distribution and the underrepresented cohort size in the oldest age group. Finally, the study did not investigate the neurophysiological changes which, according to Esch’s model, are assumed to underlie the different motivation types. Hence, they cannot be directly used for an explanation of the observed patterns. Future studies should examine, longitudinally if possible, the relationship between specific neural adaptations and changes in sources of meaning.

In order to analyze in more detail the observed differences between countries, e.g., in the importance of religion and faith, it would be interesting to replicate this study in other regions. Finally, we recommend investigating possible changes in the sources of meaning after extraordinary life events and traumatic experiences, as some authors have observed changes in the definition of life goals after such events (e.g., Triplett et al., 2012).

## Conclusion

The most important sources of meaning in the context of the German society were identified based on a representative sample of approximately 1,600 people, comprising individuals between the ages of 12 and 94 years. The results were further clustered according to four age groups to identify similarities and differences regarding perceived sources of meaning among individuals of different ages. We found that, across all age groups, aspects associated with *relationships* and *health* are most often considered as being important. For many sources of meaning, we identified age-related differences. While the importance of *partnership* increased significantly with age, the importance of *social networks* was lower in older individuals. Further age-related differences could be observed, for example, in the striving for *material possessions* (almost meaningless in Age group 4), *self-realization* and *success and career* (decrease with increasing age), *security* (most pronounced in midlife), and *peace and harmony* (significantly higher after midlife). Except for *financial security*, monetary aspects were not of great importance to the participants of this study. We interpreted the results in the light of a *neurobiological model of motivation systems*, arguing that the observed patterns are interrelated and represent the consequence of lifelong neurophysiologic adaptation processes.

## Data Availability Statement

The datasets presented in this article are not readily available because the Witten/Herdecke University’s Ethics Committee did not allow the data to be made publicly available. In the event that data is needed for review, please contact the corresponding author. Requests to access the datasets should be directed to TE, tobias.esch@uni-wh.de.

## Ethics Statement

The studies involving human participants were reviewed and approved by the Ethik-Kommission der Universität Witten-Herdecke e.V. Written informed consent to participate in this study was provided by the participants’ legal guardian/next of kin.

## Author Contributions

CK: conceptualization, investigation, formal analysis, and writing. LW: conceptualization, methodology, review, and editing. TE: conceptualization, project administration, supervision, review, and editing. All authors contributed to the article and approved the submitted version.

## Conflict of Interest

The authors declare that the research was conducted in the absence of any commercial or financial relationships that could be construed as a potential conflict of interest.

## Publisher’s Note

All claims expressed in this article are solely those of the authors and do not necessarily represent those of their affiliated organizations, or those of the publisher, the editors and the reviewers. Any product that may be evaluated in this article, or claim that may be made by its manufacturer, is not guaranteed or endorsed by the publisher.

## Footnotes

1 Karwetzky C., Werdecker, L., Michaelsen, M. M., and Esch, T. (in preparation). Happiness, satisfaction and the U-shape of life.
