# Supplementary material for: What Matters Most in Life? A German Cohort Study on the Sources of Meaning and Their Neurobiological Foundations in Four Age Groups
Source: Front Psychol. 2021 Nov 30;12:777751. doi: 10.3389/fpsyg.2021.777751 (PMC8671042; doi:10.3389/fpsyg.2021.777751)
Supplement: Supplementary file 1 [file Data_Sheet_1.docx]

Supplementary Material

**Supplementary Figure 1:** Word clouds for the youngest and the oldest age group


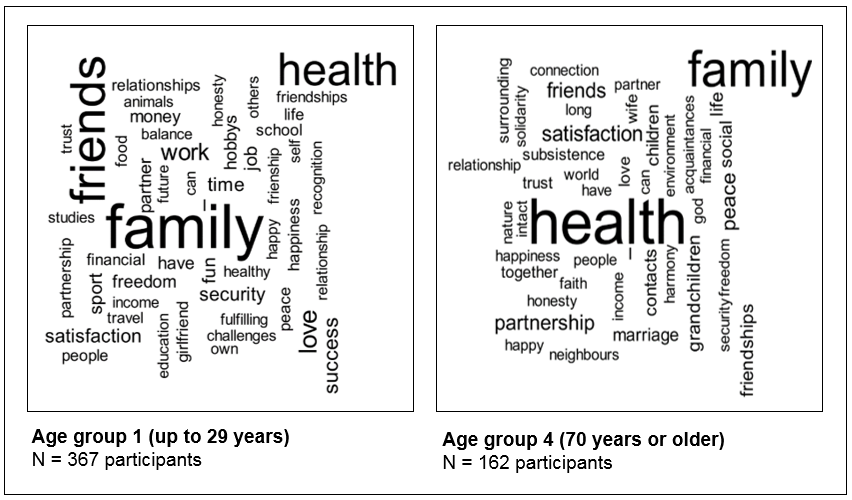


**Supplementary Figure 2:** Neurobiological model of motivation systems


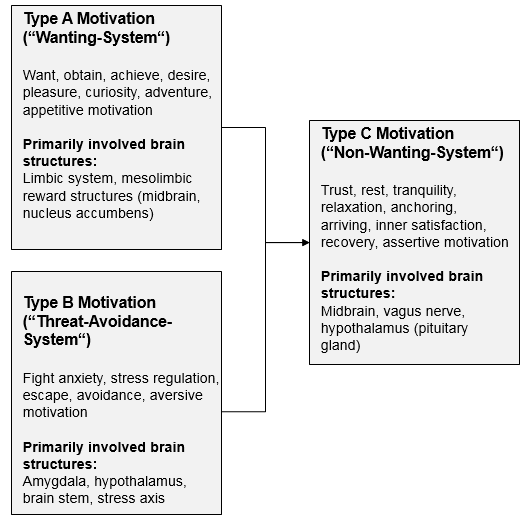


**Supplementary Table 1.** Category system

| # | **Main category** | **Sub category** | **Examples** |
| --- | --- | --- | --- |
| **1** | **Personal growth** |  | **No Codings** |
| 1.1 | Personal growth | Self-realization | e.g., personal growth, self-fulfillment, (personal) development, lifelong learning, new knowledge, acquisition of knowledge, progress, mental challenges, growth, realizing ideas, realizing dreams, development possibilities, personality development, graduating from high school, understanding science, philosophy, free access to knowledge, being able to read, understanding the world, opportunities for education |
| 1.2 | Personal growth | New experiences and challenges | e.g., variety, no routines, challenges, being open-minded, curiosity, learning a new profession, adventures, experiences, about 3 to 5 different fields of work, openness, being enthusiastic about new opportunities, learning to understand others |
| 1.3 | Personal growth | Creativity and inspiration | e.g., my own creativity, my activity, inspiration, mental stimulation, feeling power, innovation, creating something |
| 1.4 | Personal growth | Set goals (and achieve them) | e.g., a concrete vision and strategy that will move me forward, passing my driving test, ambition, motivation, personal success, goals and achievements |
| 1.5 | Personal growth | Self-knowledge / self-acceptance | e.g., to find myself, to accept myself the way I am, to understand myself, reflection, being able to see things clearly, to take life as it comes |
| 1.6 | Personal growth | Perspectives | e.g., perspectives, possibilities / options |
| **2** | **Work** |  | **e.g., profession, job, work, school (general), studies, success in my job, my independent work, job, training, professional future, own company, being good at my job, my own company, fulfilling job, fulfilling occupation Important: Combinations like “fulfilling job” were coded with "fulfillment" and “work".** |
| 2.1 | Work | Success and career | e.g., success at work, success at work, career advancement, professional future |
| 2.2 | Work | Good working conditions | e.g., good working atmosphere, positive working conditions, good working environment |
| 2.3 | Work | Joy and satisfaction at work | e.g., satisfying work, satisfaction at work, satisfaction at work, satisfying work, interesting / creative / varied / harmonious work, a good team, good job that fills me out, exciting work, pleasure in studying, that the positive outweighs the negative in my job |
| **3** | **Social rank** |  | **No codings** |
| 3.1 | Social rank | Power | e.g., power, influence |
| 3.2 | Social rank | Recognition | e.g., professional recognition, being noticed, respect, being respected, prestige, reputation, good standing, being considered competent |
| **4** | **Material possessions** |  | **No codings** |
| 4.1 | Material possessions | Money | e.g., money, wealth, luxury, prosperity, handling money, high / good / satisfactory income, sufficient money, good earnings, good livelihood  **Important:** Combinations like "money for travel" are assigned to both categories. Secure income is coded with "secure financial livelihood” |
| 4.2 | Material possessions | Good standard of living | e.g., adequate standard of living, nice living/home, comfortable living, (own) house, flat, living well, living in a nice house |
| 4.3 | Material possessions | Objects | e.g., car, clothes, technical equipment, property, sufficient material things, beautiful things, cuddly toy |
| **5** | **(Leisure) time** |  | **No codings** |
| 5.1 | (Leisure) time | To have time and spend it with what / those you love | e.g., time in general, time to spend with children, time for the things that are important to me, time to spend with beautiful things |
| 5.2 | (Leisure) time | Work-life balance | e.g., good work-life balance, good work-life balance, not working too much |
| 5.3 | (Leisure) time | Rest and relaxation | e.g., rest, retreats, no stress, (healthy) sleep, silence, calmness, no pressure to perform, relaxing within your own four walls, being able to take time out, time for inner reflection, little stress, balance between rest and action, being able to take time out |
| 5.4 | (Leisure) time | Holidays / travel | e.g., holidays, holidays with the family, travelling and getting to know and respect the country and its people, seeing a lot of the world, experiencing other places/countries, money to travel, discovering the world, Africa |
| 5.5 | (Leisure) time | Activity / entertainment / hobbies | e.g., mental and physical activity, hobby, being able to move (physically and mentally), rowing, yoga, dancing, cycling, mountain climbing, making music, being able to do sports, sports, scouts, music, TV, literature, PC games, art, cultural events, culture, artistic creation, heavy metal, philosophy |
| **6** | **Freedom** |  | **e.g., freedom, freedom of movement** |
| 6.1 | Freedom | Independence and self-determination | e.g., independence, being able to do what I enjoy doing, feeling that health challenges are manageable, being autonomous, my free spirit, mobility, freedom of choice, free choice of lifestyle, not being a burden on my environment, independence, having the choice |
| 6.2 | Freedom | Opportunities for change | e.g., scope for action, knowing and using my opportunities |
| 6.3 | Freedom | Freedom of expression | e.g., being able to express opinions freely |
| **7** | **Security** |  | **No codings** |
| 7.1 | Security | Secure financial livelihood | e.g., secure income, regular income, (a certain) material independence / security, financial security, social security, work you can live on, not having financial worries, getting by with what you have, social security, being able to provide yourself with enough food, clothing and housing, having enough food and drink, securing my basic material existence (housing, clothing, food, health care), not being afraid to exist, financial independence, securing my existence |
| 7.2 | Security | Safe workplace | e.g., safe work, occupational safety, a permanent job, stable professional situation |
| 7.3 | Security | Safe home | e.g., a roof over your head, a safe home |
| 7.4 | Security | Stability | e.g., a regulated life, ability to plan, routines, a certain security for the future, that everything remains the way it is |
| **8** | **Health / well-being** |  | **No codings**  **The sole mention of "health" is evaluated as if the person wishes health for himself or herself** 🡪 **coding with "health for me" (**🡪 **same procedure for happiness and satisfaction!)** |
| 8.1 | Health / well-being | Health for me | e.g., health, well-being / well-being for me, mobility, living without pain, maintaining the ability to work, emotional stability, getting well soon, being able to read, clear consciousness |
| 8.2 | Health / well-being | Health for us or others | This category was coded, if the answer indicates that the health of other people is meant as well:  e.g., health for my beloved people, that my baby is born healthy, well-being for my family / friends **Important:** Health for my family is coded as "health for us or others" and "family". We proceeded in the same way with "health for friends" etc. |
| **9** | **Good living environment** |  | **e.g., beautiful living environment, my environment, environment** |
| 9.1 | Good living environment | Peace and harmony | e.g., harmony, domestic peace, no war, peaceful living together, no quarrelling, social peace |
| 9.2 | Good living environment | Nature and sustainability | e.g., contact with an intact nature, stay in nature, our garden, experience nature, ecology, leave as little waste as possible, use energy as efficiently as possible, no animal testing, waves, sustainability |
| 9.3 | Good living environment | Societal security | e.g., security, social security, feeling safe, security in Bavaria, protection, safe living environment |
| 9.4 | Good living environment | A society worth living in | e.g., justice, democracy, fairly distributed prosperity, good environment, no more temporary work, little discrimination, social change, amenities in public life, freedom of expression, politics, finally clear decisions in politics, equality of all people, good people, fair treatment of all people |
| 9.5 | Good living environment | Solidarity and humanity | e.g., helpfulness, helping people financially, support, being useful to others, dealing with people who are not doing so well, support, consideration, charity |
| 9.6 | Good living environment | Understanding / tolerance | e.g., understanding, tolerance, accepting others the way they are |
| 9.7 | Good living environment | Home / roots / identity | e.g., rootedness, homeland, preservation of the homeland, preservation of our Christian society, people, Germany |
| 9.8 | Good living environment | Diversity of life | e.g., diversity, diversity of life |
| **10** | **Happiness** |  | **No codings**  **The sole mention of "happiness" is evaluated as if the person wishes for happiness for himself or herself** 🡪 **coding with "happiness for me".** |
| 10.1 | Happiness | Happiness for me | e.g., happiness, being happy, my happiness, happy moments, joy, fun, laughing, authentic moments, enjoying beautiful moments, beautiful experiences, happiness, humor, feeling well, authentic moments, joy of life, wow moments |
| 10.2 | Happiness | Happiness for us and others | This category was coded, if the answer indicates that the happiness of other people is meant as well.  e.g., that the people I am responsible for are happy, happiness for my loved ones, happiness for the family **Important:** "Happiness for my family" was coded with "Happiness for us and others" and "family |
| 10.3 | Happiness | Pleasure and passion | e.g., enjoying life, enjoyment, a fulfilled sex life, cuddling, human closeness, sexuality |
| **11** | **Live satisfaction** |  | **No codings.**  **The sole mention of "satisfaction" is evaluated as if the person wishes for satisfaction for himself or herself** 🡪 **coding with "satisfaction for me".** |
| 11.1 | Life satisfaction | Satisfaction for me | e.g., my satisfaction, inner satisfaction, inner balance, being in harmony with myself, peace of mind, inner peace, inner peace, feeling good **Important:** "no stress" and "little stress" were coded with "rest and relaxation” |
| 11.2 | Life satisfaction | Satisfaction for us or others | This category was coded, if the answer indicates that the satisfaction of other people was meant as well. e.g., satisfaction for my children, satisfaction in the family, that my son is satisfied, satisfaction not only for me **Important:** Combinations like “satisfaction of our family" were coded with "satisfaction for us or others" and "family |
| **12** | **Values** |  | **e.g., having a clear conscience, loyalty, having courage, my convictions, that my environment shares my values** |
| 12.1 | Values | Honesty | e.g., honest and open criticism, authenticity, living authentically, having honest people in my environment (also coded with "social network") |
| 12.2 | Values | Carefreeness / balance | e.g., absence of feelings such as fear, doubt, anger, loss, sadness or loneliness, patience, light-heartedness **Important:** "Balance between work and private life" was coded with “work-life balance” |
| 12.3 | Values | Reliability | e.g., reliability, being able to count on people, a reliable circle of friends (also coded with “social network”), reliable colleagues, reliable friends **Important:** Combinations like “reliable friends / colleagues / etc.” were coded with “Reliability” and e.g., “social network” |
| 12.4 | Values | Mindfulness | e.g., enjoying the moment, living a conscious life, being aware of beautiful things and enjoying them, appreciating the little things in life, paying attention |
| 12.5 | Values | Self-love and self-awareness | e.g., I, doing enough for myself, my self-esteem, self-confidence, not forget myself |
| 12.6 | Values | Gratitude / humility | e.g., gratitude, living a simple life |
| 12.7 | Values | Optimism | e.g., positive thinking, optimism, positive people (in my life), hope |
| 12.8 | Values | Friendliness | e.g., being friendly to people, friendly interaction with each other |
| 12.9 | Values | Open-mindedness | e.g., openness, being enthusiastic about things, getting to know people behind their façade, learning to understand others |
| 12.10 | Values | Responsibility | e.g., (financial) responsibility for myself, personal responsibility, sense of responsibility towards people and nature |
| 12.11 | Values | Compassion | e.g., compassion, empathy |
| 12.12 | Values | Will | will |
| 12.13 | Values | Pride | pride |
| **13** | **Sense and meaningfulness** |  | **e.g., sense, being a good mother to my child, a task in life, experiencing sense, vocation** |
| 13.1 | Sense and meaningfulness | Fulfillment | e.g., a fulfilling life, fulfillment, inner richness, fulfilling relationships / partnership, a fulfilling job / task, projects and activities in everyday life, fulfilling employment, creating something (e.g., craft activity), fulfilling professional activity, fulfillment at work, demanding work, work that fills me out **Important:** Combinations like fulfillment at work or fulfilling partnership were coded with "fulfillment" and "job" / "partnership" etc. |
| 13.2 | Sense and meaningfulness | Faith / spirituality | e.g., faith in Jesus Christ, religion, connection to God, being able to feel God's guidance, spiritual growth, spiritual security, meditation, inspiring people, being inspired |
| 13.3 | Sense and meaningfulness | Social commitment | e.g., making a contribution to society, social commitment, voluntary work, political participation, my fire brigade |
| 13.4 | Sense and meaningfulness | Effect | e.g., effectiveness, the feeling of being able to make a difference, leaving footprints, having a positive influence, self-efficacy, making the world a better place, passing on my ideas of ethics, resonance, being important, being needed |
| **14** | **Relationships** |  | **e.g., good relationships, the most important thing in my life are loving relationships, my relationships with the people who are important to me** |
| 14.1 | Relationships | Family | e.g., family cohesion, children, mother, daughter, grandchildren, having children, family happiness, connections within the family, good relationship with my relatives, family happiness, well-being of children **Important:** - Combinations like “happiness in marriage” were coded with “family” and “happiness for us or others”.  - Several mentions of the same category (e.g., mother, daughter, partner) were coded only once (applies to all categories) |
| 14.2 | Relationships | Social network | e.g., friendship, social environment, friendships, friends, nice colleagues, my class, social environment, regulars' table, scouts, positive people in my life, human warmth, reliable circle of friends (also reliability!), solving tasks together with others, social contact at eye level, the flat share in which I live, people who are close to me,  **Important:** Combinations like "reliable friends" are mentioned in both categories “reliability” and “social network”. |
| 14.3 | Relationships | Partnership | e.g., my partner, that my marriage will last long, no long life without my partner, happy partnership, growing old together |
| 14.4 | Relationships | Love and trust | e.g., love, closeness, attachment, (deep) connection, deep social bonds, balance between independence and security, human warmth, affection, experiencing emotions, trust |
| 14.5 | Relationships | Pets | e.g., my dog, cat, etc. |
| 14.6 | Relationships | Communication | e.g., spiritual exchange, good conversations, exchange with other people, deep conversations |
| 14.7 | Relationships | Participation and community | e.g., spiritual participation, intellectual participation, affiliation, participation in society, cohesion (general), community, togetherness, contact, contact with loved ones, not to be lonely, interaction |
| **15** | **A long life** |  | **e.g., a long life, lifetime, growing old** |
| **16** | **Other** |  | **No codings** |
| 16.1 | Other | Food and nutrition | e.g., balanced diet, veganism, ice cream, eating out regularly |
| 16.2 | Other | Good weather | e.g., sunny weather, warm weather, sun |
| 16.3 | Other | Future | e.g., good future for children and grandchildren, a secure future, future |
| 16.4 | Other | Positive memories | e.g., positive memories, memories |
| 16.5 | Other | Beautiful body | e.g., beautiful body, good figure, tattoos, my appearance, good body feeling |
| 16.6 | Other | Truth | truth |
| 16.7 | Other | Allowing chaos and order | allowing chaos and order |
| 16.8 | Other | Strength | strength |
| 16.9 | Other | Short distance to work | short distance to work |
| 16.10 | Other | Self-evidence | self-evidence |
